# The *Arabidopsis* Receptor-like Kinase CAP1 Promotes Shoot Growth under Ammonium Stress

**DOI:** 10.3390/biology11101452

**Published:** 2022-10-02

**Authors:** Qingye You, Nannan Dong, Hong Yang, Fang Feng, Yifei Xu, Chong Wang, Yilan Yang, Xiaonan Ma, Ling Bai

**Affiliations:** State Key Laboratory of Crop Stress Adaptation and Improvement, School of Life Sciences, Henan University, Kaifeng 475004, China

**Keywords:** ammonium toxicity, CAP1, *Arabidopsis*, cell expansion, ROS

## Abstract

**Simple Summary:**

Understanding the underlying mechanisms of NH_4_^+^ toxicity is essential for improving nitrogen use efficiency. Although numerous genes and factors have been identified to function in modulating the response to NH_4_^+^ stress, NH_4_^+^ toxicity remains poorly characterized. Our work reported here demonstrated a new role for CAP1 in shoot growth in response to NH_4_^+^ stress. The enhanced sensitivity of the *cap1-1* mutant to NH_4_^+^ stress is linked with the role of CAP1 in regulation cell wall loosening and ROS accumulation.

**Abstract:**

High levels of ammonium (NH_4_^+^) in soils inhibit plant growth and nitrogen utilization efficiency. Elucidating the underlying mechanisms of NH_4_^+^ toxicity is essential for alleviating the growth inhibition caused by high NH_4_^+^. Our previous work showed that [Ca^2+^]_cyt_-associated protein kinase 1 (CAP1) regulates root hair growth in response to NH_4_^+^ in *Arabidopsis thaliana*, and the *cap1-1* mutant produces short root hairs under NH_4_^+^ stress conditions. However, it is unclear whether CAP1 functions in other physiological processes in response to NH_4_^+^. In the present study, we found that CAP1 also plays a role in attenuating NH_4_^+^ toxicity to promote shoot growth. The *cap1-1* mutant produced smaller shoots with smaller epidermal cells compared with the wild type in response to NH_4_^+^ stress. Disruption of CAP1 enhanced the NH_4_^+^-mediated inhibition of the expression of cell enlargement-related genes. The *cap1-1* mutant showed elevated reactive oxygen species (ROS) levels under NH_4_^+^ stress, as well as increased expression of respiratory burst oxidase homologue genes and decreased expression of catalase genes compared with the wild type. Our data reveal that CAP1 attenuates NH_4_^+^-induced shoot growth inhibition by promoting cell wall extensibility and ROS homeostasis, thereby highlighting the role of CAP1 in the NH_4_^+^ signal transduction pathway.

## 1. Introduction

Plant growth is closely related to the availability of mineral nutrients, particularly nitrogen (N) [1]. To produce 1 kg of dry biomass, most non-legume plants need to take up 20–50 g of N [2]. However, N in soil is often insufficient to support optimum plant growth, and therefore, N fertilizer is commonly applied in cropping systems.

The two major forms of inorganic N in the soil are nitrate (NO_3_^−^) and ammonium (NH_4_^+^). Large quantities of N fertilizer (NH_3_/NH_4_) have been applied to agricultural soils to improve crop yields, which results in NH_4_^+^ toxicity. The typical concentration of NH_4_^+^ reported in soils ranges from 2 to 20 mM, and is even higher in some cases (40 mM) [3,4]. Although NH_4_^+^ requires less energy for assimilation than NO_3_^−^, the toxicity caused by excessive NH_4_^+^ (millimolar concentrations) is a big problem in agriculture [5,6]. For most plants, the application of NH_4_^+^ as the sole N source causes severe growth suppression. NH_4_^+^ toxicity commonly results in root and shoot growth inhibition, which includes biomass reduction, oxidative stress with overproduction of reactive oxygen species (ROS), and disturbance of pH gradients or ionic imbalances [5,6,7,8,9]. To address this agronomic problem, it is essential to thoroughly understand the mechanisms of NH_4_^+^ toxicity.

Several mechanisms that are mainly dependent on the physiological process have been elucidated, including rhizosphere acidification, energy consumption for transmembrane cycling of NH_4_^+^, ion imbalance, hormone alterations, and oxidative stress [5,10]. Furthermore, the role of NH_4_^+^ as a photophosphorylation uncoupler impairs photosynthetic processes [11,12].

ROS have important roles in the response to various environmental stresses, and high levels of ROS induce oxidative defense responses and programmed cell death [13,14,15]. ROS participate in NH_4_^+^ toxicity, as elevated levels of ROS in response to high NH_4_^+^ trigger a series of molecular pathways [16,17]. Both roots and leaves of *Arabidopsis thaliana* overproduce ROS in response to excess NH_4_^+^, and these ROS are responsible for the high-NH_4_^+^-induced growth inhibition [7,18,19].

ROS can be produced through many routes [20], and NADPH oxidases are a key source of ROS production. Plant NADPH oxidases are also called respiratory burst oxidase homologues (RBOHs) [21,22]. *A. thaliana* has 10 RBOHs (A-J); these plasma membrane-localized enzymes catalyze the formation of oxygen radicals (O_2_^−^) in the apoplast, and then, O_2_^−^ is transformed into hydrogen peroxide (H_2_O_2_) by an enzymatic dismutation step. Many abiotic and biotic stresses induce the expression of RBOH genes, such as anoxia/hypoxia, drought, and N stress [21,23]. NADPH oxidases may function as the source of ROS in NH_4_^+^-stressed plants.

To relieve the deleterious effects of ROS, the activities of ROS scavenging enzymes can be up-regulated, such as catalase (CAT), glutathione reductase (GR), and superoxide dismutase (SOD) [24,25,26,27]. Moreover, high NH_4_^+^ levels alter the apoplastic pH [28], up-regulating the activities of cell wall peroxidases (PODs) [29], which can also scavenge ROS.

A practical approach to understanding the mechanisms of NH_4_^+^ toxicity is the isolation and characterization of NH_4_^+^-responsive mutants. To explore the underlying mechanism of NH_4_^+^-triggered ROS accumulation, forward genetic screens have been used to identify genes involved in modulating ROS levels in leaves under NH_4_^+^ stress, including *ammonium overly sensitive 1* (*AMOS1*)/*ethylene-dependent, gravitropism-deficient, and yellow-green-like protein 1* (*EGY1*) and *ammonium tolerance 1* (*AMOT1*)/*ethylene insensitive 3* (*EIN3*) [19,30].

NH_4_^+^-mediated suppression of growth may be attributable to repression of cell expansion, which is associated with altered expression of cell wall modifying genes. The expansins (EXPs) and endopolygalacturonases (PGs) genes are related with cell wall assembly, members of these family have been proposed function in growth regulation to NH_4_^+^ [29]. EXPs modulate cell extensibility, they loosen the adhesion between cellulose and hemicelluloses, which lead to cell wall relaxation. EXPs are of greater importance for controlling the loosening of cell wall polymers during cell expansion. PGs are a kind of pectin-hydrolyzing enzymes which also function in cell wall relaxation by hydrolyzing the pectin network.

Receptor-like kinases have been showed function in regulating cell growth to environmental signal. Members of *Catharanthus roseus* receptor-like kinase (CrRLK) are highly expressed in elongating tissues and they have been suggested to play role in regulation cell expansion growth [31]. *Feronia* (*FER*), an intensive studied CrRLK gene, encodes a cell wall sensor receptor-like kinases and is essential for cell expansion and growth [32]. The lower expression of *FER* has been observed for seedlings treated with NH_4_^+^ [29], which proposed that FER may involve in the disturbed cell growth to NH_4_^+^. Our previous work showed that the receptor-like kinase [Ca^2+^]_cyt_-associated protein kinase1 (CAP1), a member of CrRLKs, is involved in NH_4_^+^-regulated root hair growth; the deficiency of CAP1 caused short and abnormal root hairs in *A. thaliana* [33]. Here, we demonstrated that CAP1 also plays a role in shoot growth under NH_4_^+^ stress. After culture for about one month in vermiculite, plants were treated with Murashige and Skoog (MS) liquid medium containing different levels of NH_4_^+^. The *cap1-1* mutants showed smaller leaves and smaller epidermal cells compared to the wild type in response to the high NH_4_^+^ treatments. Our analyses of cell enlargement- and ROS homeostasis-related gene expression, and the ROS content in response to NH_4_^+^ stress suggested that CAP1 attenuates the NH_4_^+^-inhibited shoot growth by promoting cell wall extensibility and ROS homeostasis.

## 2. Materials and Methods

### 2.1. Plant Materials and Growth Conditions

All *A. thaliana* lines used in this work were in the Col-0 background. All seeds were surface sterilized and stratified at 4 °C for 3 d in the dark, and then grown on plates with Murashige and Skoog (MS) medium containing 3% (*w*/*v*) sucrose and 0.6% agar. After growing for 7–10 days, 8–10 seedlings for each line were transplanted to a pot containing only vermiculite for further growth, three technical replicates were performed for each treatment, and every experiment repeated at least three times. During this period, same volume solutions containing different concentrations of NH_4_^+^ were applied to the respective plants for about 4 weeks followed by analysis. The growth conditions were 22 °C and the fluency rate of white light was ~80–100 μmol m^−2^s^−1^. The photoperiod was 16 h/8 h dark. 

Media used in this work are all modified from the MS medium. The nitrogen in MS medium consists of 37.6 mM NO_3_^−^ (KNO_3_) and 20.6 mM NH_4_^+^ (NH_4_Cl). The 4 different media we used were named according their NH_4_^+^ concentration. The first only consists of 37.6 mM NO_3_^−^ (KNO_3_), so we nominated it as 0 mM. The second is the MS medium, as above, we nominated it as 20.6 mM. The third and the fourth are the MS medium supplemented with 41.2 mM NH_4_^+^ or 61.8 mM NH_4_^+^ (NH_4_Cl), correspondingly, these two were designated as 41.2 mM or 61.8 mM.

### 2.2. Leaf Area and Epidermal Cell Area Measurements

To investigate the different shoot growth, we first measured the leaf area of the wild type and the *cap1-1* mutant under different concentrations of NH_4_^+^. The 3rd leaves were scanned, and leaf area were measured by Image J.

In order to analyze the development of leaf cells, we stained the leaf cells with FM4-64. The 3rd leaves were placed in distilled water containing 4 μM FM4-64 at room temperature for 5–10 min in the dark. Images were acquired with a confocal microscope (LSM710; Zeiss), and the epidermal cell area was measured by Image J.

### 2.3. Gene Expression Analysis

For analyzing the different leaf epidermal cell expansion between wild-type and *cap1-1* plants, *EXP1* and *EXP17*, *PG1* and a putative *PG* gene (*P-PG*, At2g43890), and *THESSEUS1* (*THE1*) and *FER* genes were selected, which have been reported that may function in ammonium regulated cell expansion [29]. The 3rd leaf of WT and *cap1-1* plants were harvested, and the relative transcription levels were quantified by quantitative real-time PCR (qRT-PCR). 

To compare the ROS related genes expression to NH_4_^+^ stress in wild type and *cap1-1*, expression of respiratory burst oxidase homologues (*RBOHs*) and catalase genes (*CAT1, CAT2*, and *CAT3)* were analyzed. Seedlings grown on MS medium for 5–7 days were transplanted to NH_4_^+^-free MS medium for 2 days. Then the seedlings were treated with 3 mM NH_4_Cl for 30 min, and about 50–100 mg seedlings were collected.

Total RNA was extracted using the TRIzol Reagent (Invitrogen) in accordance with the manufacturer’s protocol. The cDNAs were used as templates in qRT-PCR using ChamQTM Universal SYBR qPCR Master Mix (Vazyme) with gene-specific primers and the internal control (UBC9). Three biological replicates and three technical replicates were performed for each treatment. The primer pairs used for qRT-PCR are shown in Table 1. The qRT-PCR analyses were performed on an ABI Step One Plus instrument.

### 2.4. DAB staining and Image Analysis

Histochemical staining of H_2_O_2_ was performed as previously described [34] with minor modifications. Leaves were vacuum infiltrated with 1 mg/mL 3,3′-diaminobenzidine (DAB) in 2 M HCl buffer, pH 3.0. Samples were incubated for 5–6 h at room temperature in the dark. Following the incubation, the DAB staining solution was replaced with bleaching solution (ethanol:acetic acid:glycerol = 3:1:1). The samples were placed in an oven (65 °C) for 15 min and then replaced with bleaching solution, again, until the chlorophyll was completely bleached. This will bleach out the chlorophyll but leave the brown precipitate formed by the reaction of DAB with H_2_O_2_. Samples were photographed with a stereomicroscope (Olympus, SZX16). Intensities of the DAB-stained zones were quantified using Image J software. All staining and image analysis procedures were repeated at least three times.

## 3. Results

### 3.1. Deficiency of CAP1 Enhances NH_4_^+^-Mediated Inhibition of Shoot Growth in A. thaliana

In a previous study, we observed that *A. thaliana* CAP1 plays a role in root hair growth in response to NH_4_^+^. In order to further explore the function of CAP1 in other tissues under NH_4_^+^ stress, we observed the phenotype of wild-type and *cap1-1* plants under different concentrations of NH_4_^+^.

**Figure 1:**
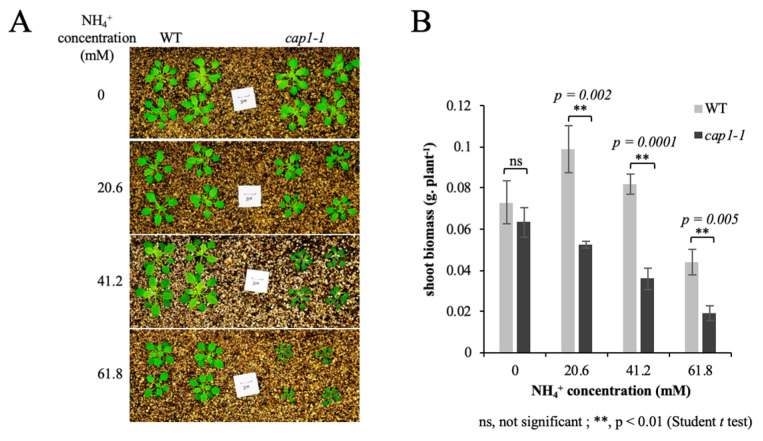
Shoot phenotypes of wild-type (WT) and *cap1-1* plants growing on media with different concentrations of NH_4_^+^. (**A**) Growth of the WT and the *cap1-1* mutant in response to different growth condition. Scale bars = 1 cm. (**B**) Shoot fresh weights of WT and *cap1-1* plants in (**A**) were averaged. Seven-day-old seedlings were transplanted to a pot containing only vermiculite. Solutions containing 0, 20.6 mM, 41.2 mM, or 61.8 mM NHCl_4_ were applied to the respective plants for about 4 weeks, and then pictures were taken. Values are the means ± SE, *n* = 8–11. (Independent samples *t*-test; ns, not significant; **, *p* < 0.01.)

After letting the plants grow for about 4 weeks, we found no obvious differences in growth between wild-type and *cap1-1* plants in the absence of NH_4_^+^ (0 mM NH_4_^+^). However, with MS medium (20.6 mM NH_4_^+^), we observed a reduction in the *cap1-1* shoot size, and an increase in the wild-type shoot (Figure 1A). When the NH_4_^+^ concentration of the MS medium was increased from 20.6 mM to 61.8 mM, the shoot size of both the wild-type and *cap1-1* plants decreased. We found significant differences in shoot growth between these two genotypes as the NH_4_^+^ concentration of the medium increased. The average fresh weight of *cap1-1* shoots dropped from about 0.063 g/plant under the NO_3_^−^ condition (0 mM NH_4_^+^) to 0.019 g/plant at 61.8 mM NH_4_^+^, while that of the wild type decreased from 0.073 to 0.044 g/plant, respectively (Figure 1B). The inhibition of shoot growth is a classic symptom of NH_4_^+^ toxicity. The significant reduction of shoot growth under NH_4_^+^ stress in the *cap1-1* mutant suggested that the disruption of CAP1 caused hypersensitivity to NH_4_^+^.

### 3.2. The cap1-1 Mutant Produces Smaller Leaves under High NH_4_^+^

**Figure 2:**
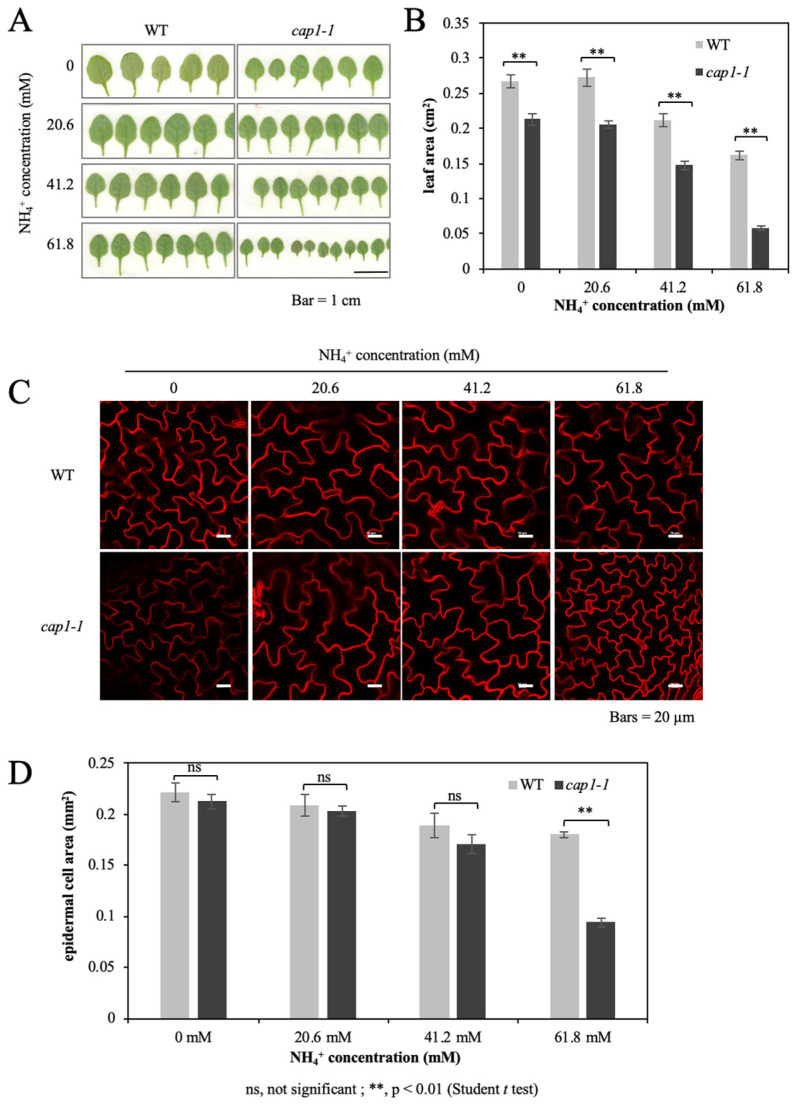
Leaf area and cell size of WT and *cap1-1* plants under different growth concentrations. (**A**) The 3rd leaf phenotype and (**B**) leaf area in WT and *cap1-1* plants grown on 4 different media with 0, 20.6, 41.2, and 61.8 mM NH_4_^+^. (**C**) The epidermis of the 3rd leaf was stained with FM4-64 and scanned by a confocal microscope. (**D**) Epidermal cell area of the 3rd leaf under MS medium without ammonium (0 mM NH_4_^+^), MS medium (20.6 mM NH_4_^+^), and MS medium with 41.2 or 61.8 mM NH_4_^+^ treatment. Seven-day-old seedlings were transplanted to a pot containing only vermiculite. Solutions containing 0, 20.6, 41.2, and 61.8 mM NHCl_4_ were applied to the respective plants for about 4 weeks. Values are the means ± SE, *n* = 8–11. (Independent samples *t*-test; ns, not significant; **, *p* < 0.01.)

The above experimental results showed that the absence of CAP1 enhances the NH_4_^+^-mediated inhibition of plant growth. We further measured the leaf area. When the NH_4_^+^ concentration was increased to more than 41.2 mM, the area of the 3rd leaf of both the wild type and the *cap1-1* mutant decreased, and the decrease in the *cap1-1* mutant was more drastic; the leaf size of the *cap1-1* mutant was about 69% of that of the wild type at 41.2 mM, and only 36% at 61.8 mM (Figure 2A,B). Moreover, leaf area for wild type showed no distinct difference between the treatments of MS medium and the MS medium without ammonium (0 mM NH_4_^+^), and the same for *cap1-1* mutant (Figure 2B).

FM4-64 is a kind of membrane dye, which can emit high intensity fluorescence after specific binding with plasma membrane. We stained the 3rd leaf with FM4-64 to visualize the size of epidermal cells (Figure 2C). After taking pictures under the fluorescence microscope, we measured the epidermal cell area. No significant difference in single epidermis cell area was found for wild type and *cap1-1* plants grown on MS and the NH_4_^+^-free medium (Figure 2D), suggesting that the deletion of NH_4_^+^ do not influence the leaf epidermal cell grow. However, difference between both lines was observed from the treatment with 41.2 mM NH_4_^+^, and substantial difference was displayed for plants grown on 61.8 mM NH_4_^+^ (Figure 2D). The average single epidermis cell area of the wild type decreased from 0.21 mm^2^ in the MS condition to 0.18 mm^2^ when treated with 61.8 mM NH_4_^+^, whereas that of the *cap1-1* mutant decreased from 0.20 mm^2^ to 0.09 mm^2^, respectively (Figure 2D). *cap1-1* leaf cell expansion is more sensitive to high level of NH_4_^+^. The decrease of the *cap1-1* epidermis cell area was much higher than in the wild type and is a main reason for the reduced *cap1-1* shoot growth under NH_4_^+^ stress, suggesting that the involvement of CAP1 in the NH_4_^+^ suppressed leaf growth may be accomplished partially by influencing cell expansion.

### 3.3. Deficiency of CAP1 Enhanced the Downregulation of Cell Enlargement-Related Genes under NH_4_^+^ Stress

Several cell wall enlargement genes related with ammonium-influenced plant growth have been reported [29], to explore whether these genes are involved in *CAP1*-regulated leaf growth to ammonium, we harvested the 3rd leaf of plants and compared the expression of these genes in wild type and *cap1-1* by qRT-PCR. No significant difference in epidermis cell area between wild type and *cap1-1* mutant showed for plants cultured on MS medium, while a marked difference can be seen under MS medium supplemented with 61.8 mM NH_4_^+^ treatment, so plants grown on MS medium and MS medium supplemented with 61.8 mM NH_4_^+^ were analyzed.

**Figure 3:**
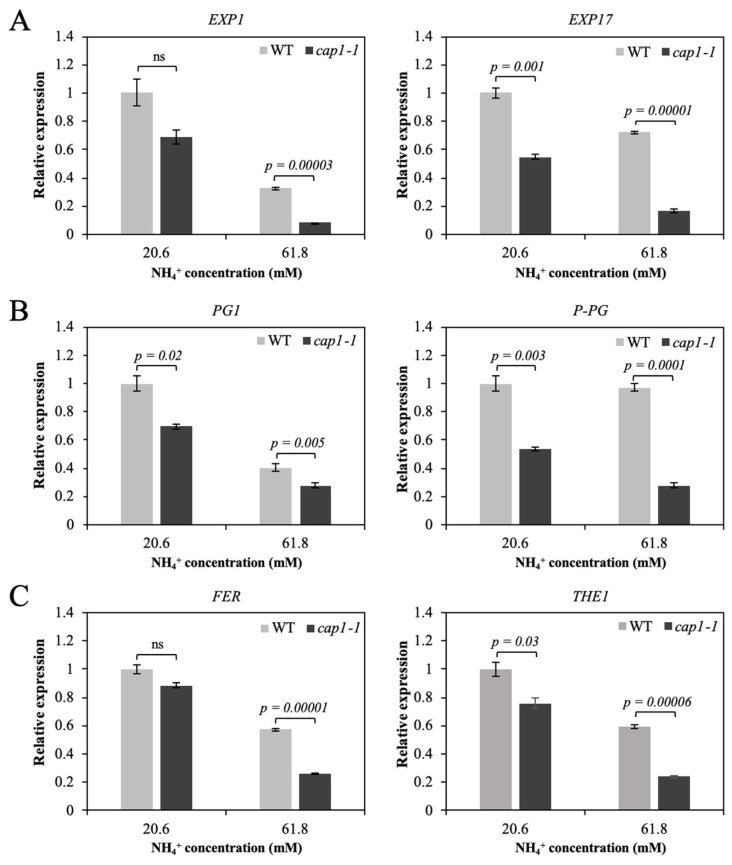
The expression of cell expansion-related genes in WT and *cap1-1* plants. Seven-day-old seedlings were transplanted to a pot containing only vermiculite with MS medium (20.6 mM NH_4_^+^) or MS medium with high content of ammonium (61.8 mM NH_4_^+^) and grew for about 4 weeks, and then 3rd leaf of *WT* and *cap1-1* plants were harvested. (**A**) Relative transcript levels for *EXP1* and *EXP17*, (**B**) *PG1* and *P-PG*, and (**C**) *THE1* and *FER* in WT and *cap1-1* plants are determined by qRT-PCR. The value for each gene in the WT at MS medium (20.6 mM NH_4_^+^) was set as 1. Data are presented as means ± SE of three replicates (independent samples *t*-test; *p* < 0.05; ns, not significant).

EXPs are engaged in cell wall loosening, the expression of *EXP1* and *EXP17* was determined. The disruption of CAP1 inhibits the expression of *EXP1* and *EXP17* (Figure 3A). In addition, as the concentration of NH_4_^+^ raised to 61.8 mM, the expression of *EXP1* and *EXP17* was declined for both lines (Figure 3A). However, more severe inhibition of both *EXP1* and *EXP17* expression was detected in the *cap1-1* mutant than in the wild type by comparing treatments between MS medium and 61.8 mM NH_4_^+^ medium. The expression of *EXP1* dropped about 68% for the wild type, and in contrast, nearly 89% for *cap1-1* mutant. In addition, the expression of *EXP17* dropped 28% for the wild type, while 71% for *cap1-1* mutant (Figure 3A). 

Expression of pectin-hydrolyzing enzymes *PG1* and *P-PG* in both wild type and *cap1-1* mutant has also been studied. Their expression was lower in *cap1-1* plants for both growth condition (Figure 3B). The transcription for *PG1* decreased about 60% for both wild type and *cap1-1* mutant by comparing 61.8 mM NH_4_^+^ condition with the MS condition. While *P-PG* decreased more in *cap1-1* plants (about 52%) than in the wild type (only about 3%) treated with 61.8 mM NH_4_^+^ (Figure 3B). The lower expression levels of these genes in the *cap1-1* mutant compared to the wild type were in accordance with the more severe impairment of shoot growth in the mutant under NH_4_^+^ stress. 

We also determined the expression of *FER* and *Thesseus1* (*THE1*), two members of the CrRLK genes. FER is thought to be associated with regulation of signaling during cell elongation. THE1 is called a cell wall integrity sensor kinase. The expression of *FER* in the *cap1-1* mutant decreased by approximately 70% under high ammonium medium (61.8 mM NH_4_^+^) compared with the MS medium condition, while in the wild type, the expression of *FER* decreased by only about 43% (Figure 3C). Similar to the expression of *FER*, the expression of *THE1* in the *cap1-1* mutant decreased by nearly 68% in the 61.8 mM NH_4_^+^ treatment compared with the MS condition, but only decreased by about 40% in the wild type (Figure 3C).

### 3.4. Deficiency of CAP1 Causes Higher NH_4_^+^-Induced ROS Levels in Shoots

**Figure 4:**
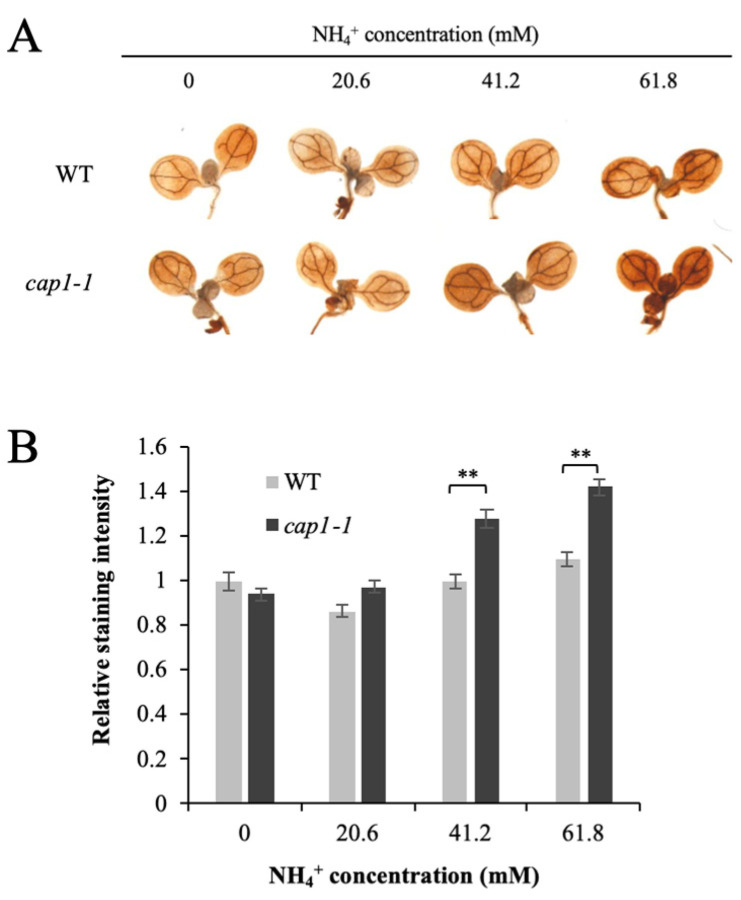
Effects of CAP1 on NH_4_^+^-induced H_2_O_2_ accumulation in shoots. (**A**) Detection of H_2_O_2_ levels in WT and *cap1-1* leaves. Seven-day-old seedlings were grown on 4 different media with 0, 20.6, 41.2, and 61.8 mM NH_4_^+^ for 3 d, and then DAB staining of shoots was performed. (**B**) The mean relative DAB staining intensity in the WT and *cap1-1* leaves in (**A**). WT grown on MS medium without ammonium (0 mM NH_4_^+^) was set as 1. Values are the means ± SE, *n* = 24–33 (independent samples *t*-test; **, *p* < 0.01).

High NH_4_^+^ induces an increase in ROS in plants. However, the biological mechanism of NH_4_^+^-induced ROS accumulation remains largely unknown. 3,3′-diaminobenzidine (DAB) staining is used to detect H_2_O_2_ in plants. We observed stronger staining in *cap1-1* shoots compared with the wild type under NH_4_^+^ treatment, and the intensity of staining increased with increased NH_4_^+^ (Figure 4A). The average DAB intensity in the *cap1-1* mutant was about 1.3-fold of the wild type with 61.8 mM NH_4_^+^ (Figure 4B). This suggested that disruption of CAP1 leads to increased accumulation of ROS under high NH_4_^+^, and conversely, that CAP1 inhibits the NH_4_^+^-induced ROS accumulation in leaves.

### 3.5. CAP1 Regulates the Transcription of AtRBOH and CAT Genes in Response to NH_4_^+^

**Figure 5:**
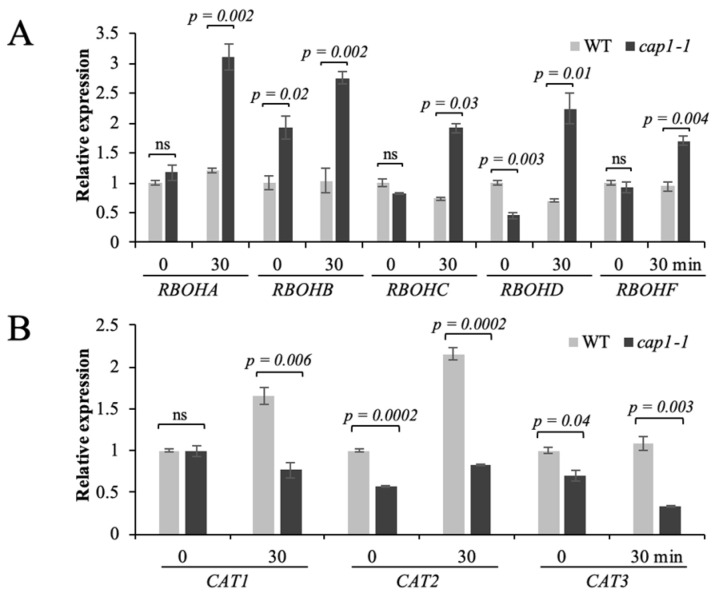
The expression of *NADPH oxidase* and *CAT* genes in WT and *cap1-1* plants to NH_4_^+^ treatment. (**A**) Relative transcript levels for *RBOHA*, *RBOHB*, *RBOHC*, *RBOHD*, and *RBOHF*, (**B**) and *CAT* genes (*CAT1*, *CAT2*, and *CAT3*) in WT and *cap1-1* plants under 3 mM NH_4_Cl treatment for 0 and 30 min quantified by qRT-PCR. Five-day-old plants grown on MS medium were transferred to MS NH_4_^+^-free medium for 2 days, then treated with 3 mM NH_4_Cl. The value for each gene in the WT at 0 min was set as 1. Data are presented as means ± SE of three replicates (independent samples *t*-test; *p* < 0.05; ns, not significant).

ROS can be generated in the apoplast via the activity of NADPH oxidases under stress [22]. Therefore, we also detected the relative transcript levels of *RBOH* genes under NH_4_^+^ stress by qRT-PCR. In the wild type, the expression of *RBOHA*, *RBOHB*, *RBOHC*, *RBOHD*, and *RBOHF* was similar at 0 and 30 min of NH_4_^+^ treatment, while their expression was significantly increased after 30 min of NH_4_^+^ treatment in the *cap1-1* mutant (Figure 5A). 

We next quantified the expression of catalase genes (*CAT1*, *CAT2*, and *CAT3*) by qRT-PCR. The expression of *CAT1* and *CAT2* increased in the wild type under NH_4_^+^ stress, but no change was detected for *CAT3*. However, in the *cap1-1* mutant, the expression of *CAT1* and *CAT3* decreased, and was significantly lower than in the wild type under NH_4_^+^ treatment (Figure 5B). Although a small increase has been observed for *CAT2* expression in the *cap1-1* mutant under NH_4_^+^ stress, it was still significantly lower than in the wild type (Figure 5B). All these results indicated that CAP1 regulates NH_4_^+^-mediated ROS accumulation through affecting the expression of *CAT* and *RBOH* genes.

### 3.6. CAP1 Transgenic Lines Suppress the Hypersensitivity of cap1-1 Mutants to NH_4_^+^

**Figure 6:**
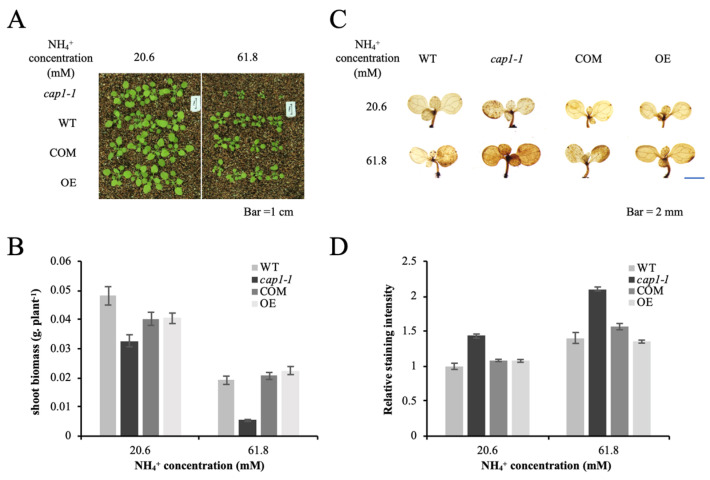
*CAP1* transgene line suppress the hypersensitivity of *cap1-1* to NH_4_^+^. (**A**) Growth of the OE, COM, WT, and the *cap1-1* mutant in response to different growth condition. Scale bars = 1 cm. (**B**) Shoot fresh weights of WT and *cap1-1* plants in (**A**) were averaged. Seven-day-old seedlings were transplanted to a pot containing only vermiculite. Solutions containing 20.6 mM and 61.8 mM NHCl_4_ were applied to the respective plants for about 4 weeks, and then pictures were taken. Values are the means ± SE, *n* = 8–11. (Independent samples *t*-test.) (**C**) Detection of H_2_O_2_ levels in WT and *cap1-1* leaves. Seven-day-old seedlings were grown on 2 different media with 20.6 and 61.8 mM NH_4_^+^ for 3 d, and then DAB staining of shoots was performed. (**D**) The mean relative DAB staining intensity in the WT and *cap1-1* leaves in (**C**). WT grown on MS medium (20.6 mM NH_4_^+^) was set as 1. Values are the means ± SE, *n* = 24–33 (independent samples *t*-test).

The enhanced shoot sensitivity to NH_4_^+^ of *cap1-1* revealed that the deletion of *CAP1* gene results this hypersensitivity. We further confirmed this by using the transgenic lines containing *AtCAP1* promoter: *CAP1* fusions (complementary lines, COM) or *35S*:*CAP1* fusions (over expression lines, OE) in the *cap1-1* background [31,33]. Both the COM and the OE lines exhibited similar shoot growth to that of the wild type under NH_4_^+^ stress, and only the *cap1-1* mutant exhibited obviously smaller shoots to higher NH_4_^+^ (61.8 mM) (Figure 6A). Average shoot biomass for both transgenic plants showed no difference compared with that of the wild type to NH_4_^+^ stress (Figure 6B). These results further proved that CAP1 plays a role in regulating shoot growth to high level of NH_4_^+^.

Furthermore, ROS levels were also studied for seedlings. DAB staining for both OE and the COM transgenic seedlings were weaker than that of the *cap1-1* mutant, whereas the staining patterns of both transgenic lines are similar as the wild type to both treatment of NH_4_^+^ (Figure 6C,D). These staining results further confirmed that the disruption of *CAP1* gene causes accumulation of ROS to NH_4_^+^ stress, that in turn suppress shoot growth.

## 4. Discussion

NH_4_^+^-mediated growth retardation is a major issue in cropping systems. The most visible phenotype of NH_4_^+^ toxicity is reduced growth of roots and leaves [5]. Genes that function in NH_4_^+^ toxicity have been discovered through mutant analysis. Studies on the Arabidopsis NH_4_^+^-sensitive mutant *hsn1* (*hypersensitivity to NH_4_^+^ 1*)/*vtc1* (*vitamin c1*) showed that the protein N-glycosylation mediated by GDP–mannose pyrophosphorylases (GMPase) may regulate root elongation in response to NH_4_^+^ stress [35,36,37]. The mutant of *AMOS1/EGY1* displays severe chlorosis under NH_4_^+^ stress. During NH_4_^+^ stress, the *AMOS1*-dependent retrograde signaling pathway, in which signals from the chloroplast modulate nuclear gene expression, is integrated with abscisic acid signaling. The elevated H_2_O_2_ induced by NH_4_^+^ was markedly lower in *amos1* seedlings than in the wild type [30]. *AMOS2* plays a role in NH_4_^+^-mediated root and shoot growth, and severely suppressed shoot biomass and inhibition of root growth were observed in the *amos2* mutant [38]. AMOS2 was reported to function in NH_4_^+^ stress mainly by controlling cation homeostasis, such as potassium (K^+^), calcium (Ca^2+^), and magnesium (Mg^2+^) [38]. AMOT1/EIN3 has been reported to function in the NH_4_^+^-induced impairment of shoot growth through positively mediating the NH_4_^+^-induced H_2_O_2_ accumulation in leaves. Enhanced tolerance to NH_4_^+^ was observed for the *amot1* mutant, which showed increased shoot growth compared to the wild type [38]. Although these genes have been implicated in NH_4_^+^ toxicity, the current understanding of their roles in NH_4_^+^ toxicity remains limited. Previous studies showed that CAP1 functions in NH_4_^+^-related root hair growth; the knockout mutant *cap1-1* displays normal root hairs only when grown on NH_4_^+^-free medium [33]. In this study, we investigated the growth of the *cap1-1* mutant under different levels of NH_4_^+^. The significantly enhanced downregulation of leaf biomass and increase in ROS levels in response to NH_4_^+^ in the *cap1-1* mutant compared with the wild type suggested that CAP1 functions in attenuating the sensitivity to NH_4_^+^ by negatively mediating NH_4_^+^-induced H_2_O_2_ accumulation.

The high levels of NH_4_^+^ inhibited *cap1-1* shoot growth (Figure 1 and Figure 2), which suggests a function of CAP1 in leaf expansion under high NH_4_^+^. Severe suppression of leaf growth in response to NH_4_^+^ has been attributed to suppression of both cell enlargement and cell division. The NH_4_^+^-mediated repression of tobacco (*Nicotiana tabacum*) leaf growth is related with both cell growth and cell number [39], while root growth suppression is more attributed to the suppression of cell growth [35,40]. In our study, the significantly smaller cell area of *cap1-1* mutant leaves suggested that cell growth is altered under NH_4_^+^ stress in *A. thaliana*. As cell growth is limited by the cell wall, cell wall loosening enables cell enlargement. One of the factors controlling cell enlargement at the individual cell level is the presence of the cell wall. Cell extensibility is closely related with the cell wall architecture. Proteins or enzymes involved in cell wall structure can modulate cell extensibility; for example, pectin hydrolyzing enzymes and EXP. Altered activities of cell wall-modifying proteins is one reason for the NH_4_^+^-triggered growth retardation in plants, and EXP and PG have been implicated in NH_4_^+^-inhibited cell growth [29]. Our results suggested that the decrease of leaf area of the *cap1-1* mutant is partly due to the decrease of cell area when exposed to NH_4_^+^ stress. The inhibited growth is related with cell wall structure, and the smaller leaf area of *cap1-1* mutant may suggest the different expression pattern for genes related with cell growth. The transcript levels of *EXP1*, *EXP17*, and two *PG* genes were markedly decreased in the *cap1-1* mutant in response to increasing concentrations of NH_4_^+^, but to a lesser extent in the wild type (Figure 3), suggesting that CAP1 plays a role in regulating cell wall extensibility in response to NH_4_^+^ by influencing the expression of these genes. The lower activity and/or expression of pectin hydrolyzing enzymes and expansins may limit cell wall expansion. Although we did not detect their protein activities in this study, the lower expression of these genes in the *cap1-1* mutant in response to NH_4_^+^ compared to the wild type suggests that they are involved in the NH_4_^+^-mediated shoot growth impairment in the mutant. 

CAP1 belongs to the CrRLK family. Other genes of this family have also been reported to participate in regulating plant growth, and plants with mutations of CrRLK family genes show growth inhibition phenotypes [31]. Among the CrRLK genes, *FER* is essential for pollen tube and root hair growth [41,42,43], and *THE1* is responsible for cell elongation [44]. The expression of both genes decreased in the wild type and *cap1-1* plants under high NH_4_^+^ (Figure 3), suggesting that *FER* and *THE1* are involved in NH_4_^+^ stress. Further, the lower expression of these genes in the *cap1-1* mutant compared with the wild type were in accordance with the markedly inhibited cell growth in the mutant (Figure 3), further suggesting that CAP1 plays an indispensable role in attenuating NH_4_^+^-inhibited cell expansion.

ROS are generated by various environmental stresses. Increased ROS in response to high NH_4_^+^ is a common symptom of NH_4_^+^ toxicity [7], and has been reported in both leaves and roots [16,24,25,45,46], which will induce oxidative defense responses. However, the mechanisms underlying NH_4_^+^-induced ROS accumulation are not clear [9,19]. AMOS1/EGY1 and AMOT1/EIN3 have been reported to function in regulating H_2_O_2_ metabolism in response to NH_4_^+^ stress [19,30]. In this study, although we observed elevated H_2_O_2_ levels in both wild-type and *cap1-1* seedlings under NH_4_^+^ stress, the *cap1-1* mutant showed markedly higher levels in our DAB staining assay (Figure 4 and Figure 6). The increased level of H_2_O_2_ in the *cap1-1* mutant suggested that CAP1 plays a role in ROS homeostasis under NH_4_^+^ stress (Figure 4 and Figure 6). As it is reported that ROS affected the transcription for cell wall remodeling enzymes [47], the perturbed ROS in the mutant may be one of the reasons that influence expression of genes is related to cell wall extensibility. However, correlation between CAP1 with the cell enlargement will need further study, the underlying mechanisms by which this process is accomplished may related with CAP1-mediated maintenance of ROS homeostasis to NH_4_^+^ stress.

A prominent source of H_2_O_2_ production is the NADPH oxidases [14]. It has been reported that expression of *RBOHD* was not induced under NH_4_^+^ stress in wild-type leaves [48]. Additionally, Li [19] found that the expression of *RBOHA*, *RBOHB*, *RBOHD*, and *RBOHF* was not induced by NH_4_^+^ in wild-type leaves. Similarly, the transcript levels of the *AtRBOH* genes investigated in the present study remained relatively unchanged in the wild type under NH_4_^+^ stress (Figure 5). However, the expression of *RBOHA*, *RBOHB*, *RBOHC*, *RBOHD*, and *RBOHF* was strongly induced in the *cap1-1* mutant after treatment with NH_4_^+^ (Figure 5). These results indicated that ROS generated by these AtRBOHs may function in NH_4_^+^ stress through a CAP1-dependent pathway. Concurrently, this induced oxidative stress can trigger ROS scavenging pathways. The relatively lower transcript levels of *CAT* genes in the *cap1-1* mutant compared with the wild type under high NH_4_^+^ also suggested the function of CAP1 in NH_4_^+^-mediated ROS homeostasis (Figure 5). The expression patterns of these genes suggested a role for CAP1 in maintaining ROS homeostasis in response to NH_4_^+^ stress by balancing ROS production and scavenging. The higher ROS content of the *cap1-1* mutant under NH_4_^+^ stress leads to more severe shoot growth reduction than in the wild type.

## 5. Conclusions

**Figure 7:**
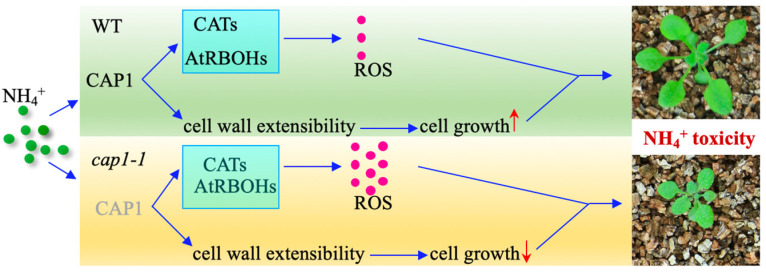
A proposed model for the role of CAP1 in shoot growth in response to NH_4_^+^ stress. Under NH_4_^+^ stress, CAP1 regulates the expression of RBOH, CAT, and cell expansion-related genes to maintain ROS homeostasis and cell wall expansion to allow shoot growth. Deficiency of CAP1 enhances the downregulation of cell expansion-related genes and the accumulation of ROS in shoots under NH_4_^+^ stress, which results in shoot growth inhibition.

In conclusion, understanding the underlying mechanisms of NH_4_^+^ toxicity is essential for improving nitrogen use efficiency. Although numerous genes and factors have been identified to function in modulating the response to NH_4_^+^ stress, NH_4_^+^ toxicity remains poorly characterized. The work reported here demonstrated a new role for CAP1 in shoot growth in response to NH_4_^+^ stress (Figure 7). The enhanced sensitivity of the *cap1-1* mutant to NH_4_^+^ stress is linked with the role of CAP1 in regulation cell wall loosening and ROS accumulation.

## Figures and Tables

**Table 1:** The primer sequences of qRT-PCR.

Gene	Primer Sequence
*EXP1* (At1g69530)	F: GAAGAGTGCCGTGCGTGAG R: TAGGTTGAAGTAAGAGTGTCCGTTT
*EXP17* (At4g01630)	F: GCCTCTGGTACAATGGGTGG R: TGAGCAAAGTTCGGTGGACA
*PG1* (At3g26610)	F: CCAATGAAACCAACGGCACT R: TGACTTTGACTCCATCCGAATC
*Putative-PG* (At2g43890)	F: ACAGGGTTCTGGAGTGAAGATTAGTC R: TCGCTTGGCACGGATTACT
*FER* (At3g51550)	F: TGCCGTCACTTCTCGTTTGC R: CTTGCTCGGACATTGGGTTG
*THE1* (At5g54380)	F: TCAAGAAGGCGGTAATGGACA R: CAACCCCAAGCAACGAACTC
*RBOHA* (At5g07390)	F: AGGGGTCGTTTGACTGGTTC R: CTCGTAAACGCTGGTGCAGT
*RBOHB* (At1g09090)	F: CGAGGTGATGGGCTACTGTG R: TGTTCCTACAAACGGGCAAG
*RBOHC* (At5g51060)	F: GAGAACAGTCAGAGAACACGTA R: TTCGTTTCAGCAAAGTAAGCTC
*RBOHD* (At5g47910)	F: GCTCCGTGCTTTCAGATCAA R: TTTGAATCCTTGTGGCTTCG
*RBOHF* (At1g64060)	F: GCCGACGAAACAACAAAGAA R: CACCAATGCCAAGACCAACT
*CAT1* (At1g20630)	F: CTGGGATTCAGACAGGCAAGA R: AACCAAACCGTAAGAGGAGCAT
*CAT2* (At4g35090)	F: CCCGTGTCTTCTCCTATGCC R: TAACCTCCTCGTCCCTGTGC
*CAT3* (At1g20620)	F: AATCACAGCCACGCCACTAA R: TCAGAACCAAGCGACCAACC
*UBC9* (At4g27960)	F: CACAATTTCCAAGGTGCTGCTATCG R: GTACATGTGAGCTATCTCAGGGACCAAA

## Data Availability

Not applicable.

## Abbreviations

CAP1: [Ca^2+^]_cyt_-ASSOCIATED PROTEIN KINASE1; N: Nitrogen; NO_3_^−^: Nitrate; NH_4_^+^: Ammonium; ROS: Reactive oxygen species; RBOHs: Respiratory burst oxidase homologues; CAT: Catalase; GR: Glutathione reductase; SOD: Superoxide dismutase; PODs: Peroxidases; AMOS1: AMMONIUM OVERLY SENSITIVE1; EGY1: ETHYLENE-DEPENDENT, GRAVITROPISM-DEFICIENT, AND YELLOW-GREEN-LIKE PROTEIN1; AMOT1: AMMONIUM TOLERANCE 1; EXP: expansins; PG: Endopolygalacturonases; CrRLKs: *Catharanthus roseus* receptor-like kinases; FER: FERONIA; THE1: THESSEUS1; DAB: 3,3′-diaminobenzidine; NUE: Nitrogen utilization efficiency; GMPase: GDP–mannose pyrophosphorylases; K^+^: Potassium; Ca^2+^: Calcium; Mg^2+^: Magnesium.
